# Deciphering the origin and therapeutic targets of cancer of unknown primary: a case report that illustrates the power of integrative whole-exome and transcriptome sequencing analysis

**DOI:** 10.3389/fonc.2023.1274163

**Published:** 2024-01-22

**Authors:** Majd Al Assaad, Nara Shin, Michael Sigouros, Jyothi Manohar, Zoia Antysheva, Nikita Kotlov, Daria Kiriy, Anastasiia Nikitina, Mikhail Kleimenov, Anastasiya Tsareva, Anastasiya Makarova, Victoria Fomchenkova, Julia Dubinina, Alexandra Boyko, Nava Almog, David Wilkes, Joanna G. Escalon, Ashish Saxena, Olivier Elemento, Cora N. Sternberg, David M. Nanus, Juan Miguel Mosquera

**Affiliations:** ^1^ Department of Pathology and Laboratory Medicine, Weill Cornell Medicine, New York, NY, United States; ^2^ Englander Institute for Precision Medicine, Weill Cornell Medicine, New York, NY, United States; ^3^ BostonGene Corporation, Waltham, MA, United States; ^4^ Department of Radiology, Weill Cornell Medicine, New York, NY, United States; ^5^ Department of Medicine, Weill Cornell Medicine, New York, NY, United States

**Keywords:** cancer of unknown primary, molecular targets, genomic testing, tumor of origin, diagnostic tools, personalized treatment, biomarkers

## Abstract

Cancer of unknown primary (CUP) represents a significant diagnostic and therapeutic challenge, being the third to fourth leading cause of cancer death, despite advances in diagnostic tools. This article presents a successful approach using a novel genomic analysis in the evaluation and treatment of a CUP patient, leveraging whole-exome sequencing (WES) and RNA sequencing (RNA-seq). The patient, with a history of multiple primary tumors including urothelial cancer, exhibited a history of rapid progression on empirical chemotherapy. The application of our approach identified a molecular target, characterized the tumor expression profile and the tumor microenvironment, and analyzed the origin of the tumor, leading to a tailored treatment. This resulted in a substantial radiological response across all metastatic sites and the predicted primary site of the tumor. We argue that a comprehensive genomic and molecular profiling approach, like the BostonGene^©^ Tumor Portrait, can provide a more definitive, personalized treatment strategy, overcoming the limitations of current predictive assays. This approach offers a potential solution to an unmet clinical need for a standardized approach in identifying the tumor origin for the effective management of CUP.

## Introduction

Cancer of unknown primary (CUP) is a diagnosis of a heterogeneous group of metastatic tumors for which no tissue of origin has been identified after extensive pathological, radiological, and clinical assessment (1, 2). The characterization of CUP to identify effective treatments that may improve patient survival continues to be challenging despite the increased accessibility to imaging, immunohistochemistry, and molecular tools. The treatment of CUP does not only require the identification of molecular targets for treatment but also the identification of the tumor of origin because the response to targeted treatment is often also dependent on cancer subtyping. An example of this is the different response to BRAF-targeted treatment in different tumors harboring the BRAF V600E mutation (3).

Currently, 3% to 5% of all cancer diagnosis is CUP, and it is the third to fourth most common cause of cancer death (4, 5). In the past five decades, there has been an observable biphasic trend in the diagnosis of CUP. Initially, there was an increase, largely attributable to the increased sensitivity of diagnostic tools, including imaging modalities. Following this, a decline was observed, which can be primarily credited to enhancements in the capabilities to detect the primary neoplastic site, including immunohistochemistry, molecular testing, and improved imaging (6). Nevertheless, survival of patients with CUP is poor with a prognosis typically less than a year, most often with empirical treatment (7). This poor prognosis is caused primarily by the advanced stage at diagnosis and resistance to empirical treatment (8, 9).

For patients with a diagnosis of CUP, molecular profiling is being developed to better characterize the tumor of origin (10). A better characterization of those tumors gives a better chance for the patients to respond to approved systemic or targeted therapies or to be enrolled in clinical trials for specific cancer types (11–13).

Several predictive assays have been developed using various techniques, including a genome-wide expression assay for predicting tumor origin, a 2000-gene classification RNA-based model (CancerTYPE ID; Biotheranostics, San Diego, CA, USA), and a 64-tissue-specific microRNA assay (Tissue of Origin; Cancer Genetics, Rutherford, NJ, USA), which are all commercially available (14–16). Further approaches in development, such as miRNA expression profiling and an assay using methylation signatures, aim to predict the tissue of origin (17, 18). However, while these developments mark progress, the clinical landscape of managing CUP is yet to be fully shaped by a standardized testing modality. The practical advantages of these methods need to be thoroughly evaluated in clinical trials, as their true impact on patient treatment is still unclear. This highlights an unmet need for a definitive, standardized approach in identifying the tumor origin for the effective management of CUP.

There is a strong belief that knowing the site of origin could improve therapeutic strategies and increase the survival of patients with responsive CUP tumor types (19). However, one clinical trial reported that there was no benefit from site-specific chemotherapy compared with empirical chemotherapy (10). Thus, more complex and molecular profiling for the determination of treatment modalities is needed for the management of patients with CUP. In addition to determining the origin of the tumor, molecular evaluation is required to identify therapeutic targets, such as hotspot mutations and mutational signatures (20, 21).

In this article, we present a successful approach using a novel genomic analysis for the evaluation and treatment of a patient with CUP. The patient had a history of multiple primary tumors, including urothelial cancer and metastatic disease without an origin after radiological and pathological evaluation. He was being treated for metastatic urothelial cancer in the lung. Whole-exome sequencing (WES) and RNA sequencing (RNA-seq) integrated analysis was done after rapid progression on empirical chemotherapy. Our approach identified a molecular target, characterized the tumor expression profile and the tumor microenvironment, and analyzed the origin of the tumor. This led to tailored treatment that resulted in substantial radiological response in all the metastatic sites and the predicted primary site of the tumor.

## Case history

**Figure 1:**
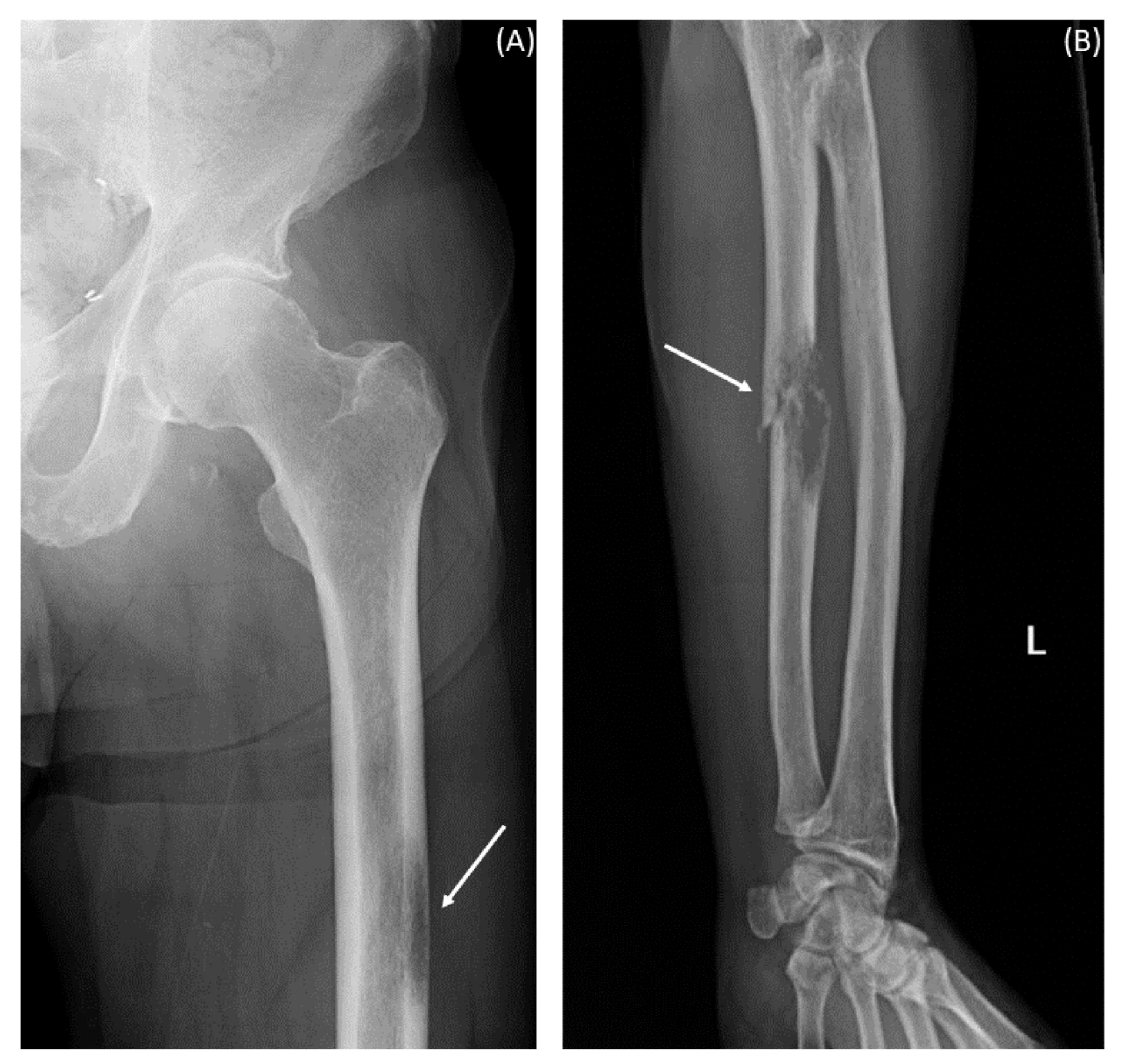
X-ray images at presentation with pain after fall. The arrows indicate hypodensities suggestive of metastatic lesions. **(A)** Left femur. **(B)** Left forearm.

An 81-year-old gentleman presented to the emergency department with an ulnar pathologic fracture and leg pain after falling at home (**Figure 1**). The patient, currently a non-smoker, has a history of 12.5 pack-years of smoking. He also had previous diagnoses of anxiety, aortic stenosis (treated with balloon valvuloplasty), hypersensitivity lung disease, hyperlipidemia, ischemic stroke, zoster, and excised melanoma (15 years prior). In addition to the balloon valvuloplasty and the melanoma excision, his surgical history included the removal of a back epidermoid cyst and umbilical and inguinal hernia repair. His family history included colorectal cancer in his mother, sudden death in his father, Raynaud’s disease in his son, Crohn’s disease in his daughter, and heart disease in his brother. He was being followed for urothelial carcinoma that was diagnosed 2 years prior to presentation. Abdomino-pelvic computed tomography (CT) scan at the time of the diagnosis of the urothelial carcinoma showed a 2.5-cm bladder lesion and a 1.5-cm left iliac lymph node, and the chest CT scan showed an irregular cystic focus of the right lung with peripheral fibrosis. A transurethral biopsy of the bladder lesion showed invasive high-grade urothelial carcinoma with undifferentiated sarcomatous features. PD-L1 immunohistochemistry (IHC) was positive with a combined positive score (CPS) of more than 50. The lung cystic lesion could not be biopsied due to the small size of the solid component and his severe emphysema. The urothelial carcinoma was managed with six cycles of neoadjuvant carboplatin and gemcitabine that resulted in interval resolution of the bladder lesion and the prominent lymph node. Radical cystoprostatectomy showed no residual invasive urothelial carcinoma (pTis pN0) with therapy-related changes and an incidental organ-confined Gleason grade 6 prostatic adenocarcinoma (pT2 pN0).

**Figure 2:**
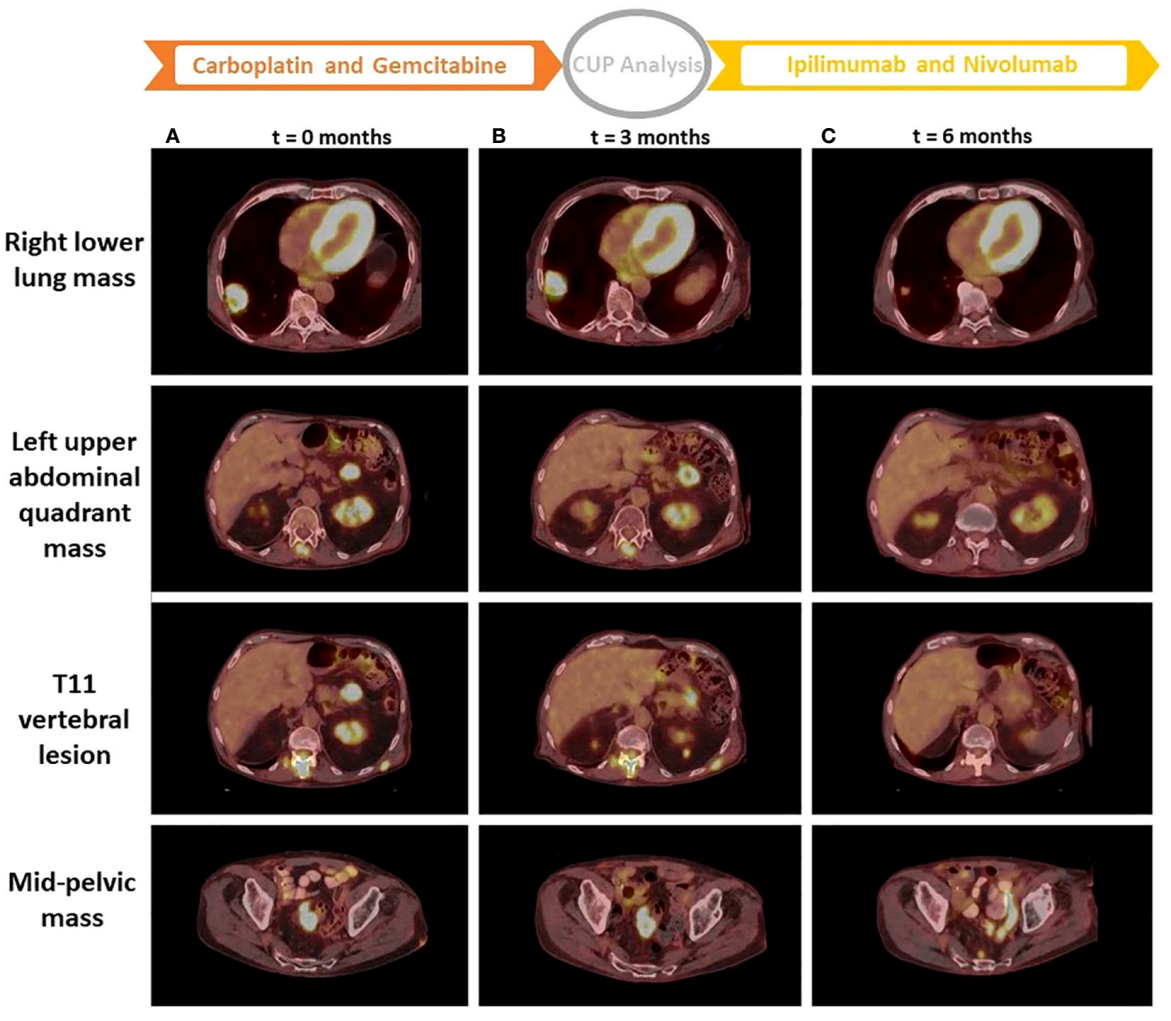
PET CT scan images from three time points. Images of column **(A)** are from a PET CT scan at the start of treatment with carboplatin and gemcitabine (*t* = 0 months). Images of column **(B)** are from a PET CT scan at the start of treatment with ipilimumab + nivolumab after performing the CUP analysis (*t* = 3 months). Images of column **(C)** are from a PET CT scan 3 months after treatment with ipilimumab + nivolumab (*t* = 6 months). *t*, time; CUP, cancer of unknown primary.

**Figure 3:**
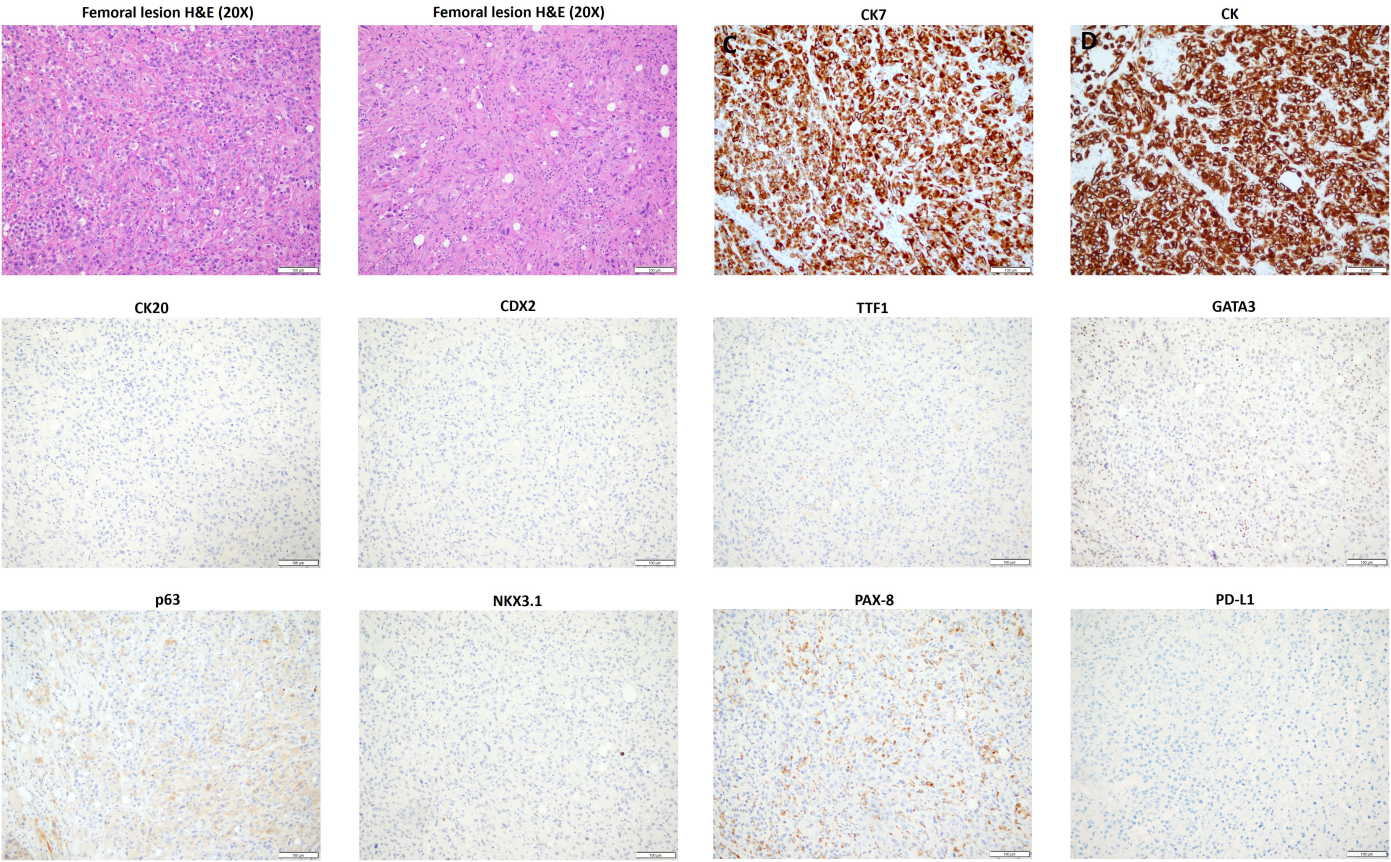
Histopathology and immunoprofile of the excised femoral lesion. H&E, hematoxylin and eosin; CK, cytokeratin.

After a year and a half of loss to follow-up to oncology, the patient presented after a fall with left forearm and leg pain. Physical exam was positive for swelling and tenderness in his left forearm. An X-ray showed a permeative osseous lesion with cortical erosion at the diaphysis of the ulna and a cortical lucency in the lateral aspect of the left femur, both concerning metastasis (**Figure 1**). A positron emission tomography (PET) CT scan showed a fluorodeoxyglucose (FDG) avid retrocaval lymph node, hypermetabolic abdominopelvic lymphadenopathy, left femoral and ulnar lesions, posterior elements of the T11 vertebral body, and a hypermetabolic lung lesion at the location of the previously seen cystic lesion (**Figure 2A**). The ulnar and femoral lesions were excised for diagnostic and treatment purposes. Histopathology showed a poorly differentiated metastatic carcinoma with sarcomatoid elements. By immunohistochemistry, the tumor was positive for pan-cytokeratin (CK) and CK7 and negative for CK20, p63, GATA3, TTF1, PAX8, NKX3.1, and CDX2 (**Figure 3**). PD-L1 immunohistochemical staining was negative (CPS = 0). This immunoprofile was diagnostic of carcinoma but did not support the origin of either colon (CDX2 negative), lung (TTF1 negative), renal (PAX-8 negative), or bladder (GATA-3 negative) primary sites. Along with tumor histomorphology, the immunoprofile also excluded metastatic malignant melanoma. While the histological and immunohistochemical profile and comparison to prior urothelial carcinoma biopsy could suggest metastatic sarcomatoid carcinoma from prior urothelial carcinoma, the site of origin could not be certainly determined. The patient was started on treatment with carboplatin/gemcitabine for what was presumed to be a metastatic urothelial carcinoma and showed an overall progression on FDG PET scan of widespread FDG avid disease (**Figure 2B**).

While the patient was on chemotherapy, further attempts were made to characterize the metastatic tumor. Thus, TruSight Oncology 500 (TSO500), a panel-based next-generation sequencing assay of the formalin-fixed paraffin-embedded (FFPE) tissue from his femoral lesion, showed KRAS (c.34G>T) and *BRAF* (c.1780G>A) mutations with high tumor mutational burden (TMB) and no microsatellite instability (MSI). TSO500 of the urothelial carcinoma showed *BRCA1* (c.3430_3458del29), *BRCA2* (c.7963C>T and c.8084C>G), *TP53* (c.273G>A), and *CDKN2A* (c.355G>T) mutations with high TMB and microsatellite-stable (MSS) status. Based on those molecular tests, the primary urothelial carcinoma and the metastatic tumor had different molecular profiles. Due to the molecular differences between the bladder and femoral tumors, combined with the uncertainty of pathological and clinical assessments, a diagnosis of CUP was made.

**Figure 4:**
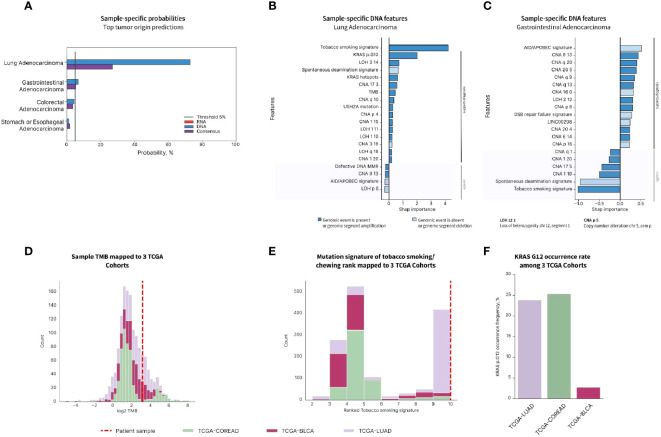
Tumor origin prediction using BostonGene Tumor of origin classifier. **(A)** Probabilities of top tumor origins predicted by DNA, RNA, and consensus classifiers. Feature importance plots provided by the DNA classifier for lung adenocarcinoma **(B)** and gastrointestinal adenocarcinoma **(C)** show molecular events with major roles in tumor origin prediction. Distributions of TMB **(D)**, tobacco smoking mutation signature rank **(E)**, and *KRAS G12* mutation rate **(F)** among the three TCGA cohorts: colorectal (TCGA-COREAD), urothelial (TCGA-BLCA), and lung adenocarcinoma (TCGA-LUAD). The patient’s value is represented with a dashed line. The data presented has been elaborated based on the commercially available service.

To better characterize the metastatic disease and to find the best management, the patient was enrolled in a prospective study for CUP patients (IRB protocol 20-05022007). Frozen tissue specimen from the femoral metastatic lesion was extracted for DNA and RNA that was used for WES and RNA-seq. BostonGene^©^ Tumor Portrait was established from the analysis of WES and RNA-seq. To produce an unbiased molecular profile, the analysis was blinded to the clinical, pathological, IHC, and radiological data. The results of the tumor profiling were then analyzed in the context of the clinical testing done. The tumor profiling confirmed the *BRAF* and *KRAS* mutations and it showed high TMB. BostonGene Tissue of origin classifier trained and validated on both genomic and transcriptomic molecular patterns was applied to predict the tumor origin of the sample and clearly favored a lung adenocarcinoma with the highest probability of 72.8% (**Figure 4A**). The top second prediction with a much lower probability was gastrointestinal (6.9%), consisting of colorectal (4.5%) and stomach (1.2%) (**Figure 4A**). The molecular events that played a major role in lung adenocarcinoma prediction were the presence of tobacco smoking mutational signature performed independently (22), *KRAS* G12 somatic mutation, TMB, and several characteristic copy-number alterations (CNAs) (**Figure 4B**), whereas for GI adenocarcinoma, only CNA features were in favor of diagnosis (**Figure 4C**). Indeed, when compared with the distributions of TMB (**Figure 4D**) and smoking signature rank (**Figure 4E**) and frequencies of *KRAS* G12 (**Figure 4F**) among The Cancer Genome Atlas (TCGA) cohorts, the sample showed the highest similarity with the TCGA-LUAD (lung adenocarcinoma) cohort rather than the TCGA-COREAD (colorectal adenocarcinoma) and TCGA-BLCA (bladder carcinoma). RNA-seq showed a high expression of PD-L1. Although a gastrointestinal tract primary could not be ruled out by the blinded Tumor Portrait, it was low on the list of clinical differentials because IHC of the metastatic tumor was CK20 and CDX2 negative, and on radiology, there was a low suspicion of a gastrointestinal tract primary tumor.

By accounting for all the results of clinical and molecular profiling of the tumor, a multidisciplinary discussion led to stopping the treatment with carboplatin and gemcitabine. The patient was started on treatment with nivolumab plus ipilimumab for a highly probable primary non-small cell lung cancer (NSCLC) with high TMB and negative PD-L1 (23). Other observations such as high RNA expression of PD-L1 (24) and a high degree of tumor infiltration by immune cells suggested a more favorable prognosis for the patient undergoing this treatment (25, 26). The patient was treated with three cycles of ipilimumab and nivolumab and experienced no adverse effects. A follow-up PET CT scan showed an overall partial response to treatment based on the Response Evaluation Criteria in Solid Tumours (RECIST) 1.1 criteria. The abdominal and mid-pelvic masses have demonstrated a decrease in size with a resolution of FDG uptake (**Figure 2C**). Also, increased sclerosis and resolved FDG uptake of osseous metastases including T11 vertebral metastasis (**Figure 2C**) further indicated treatment response.

## Discussion

Cancer of unknown primary is a diagnosis of exclusion. It is a metastatic disease with poor differentiation and an unspecific immunohistochemical profile (2, 9). Multiple assay-based panels that used real-time PCR, RNA-seq, and methylation studies have been developed to identify the tumor of origin. Both the FDA-approved panels and the experimental panels for tumor of origin identification have shown good accuracy but present questionable clinical implications. Finding the tumor of origin does not imply a better management strategy or better response to treatment (3, 10). Also, the reported accuracy of the developed panels has two major flaws. First, cancers of *known* primary used as control cases for these panels have better differentiation and more specific molecular profiles compared with CUP cases (8, 9). Second, there is no available standard assay to determine the accuracy of the panels in testing CUP cases (13). The BostonGene^©^ Tumor Portrait is a comprehensive genomic and molecular profiling report. It integrates WES and RNA-seq and offers several key analyses: determining the tumor of origin, identifying potential treatment targets, characterizing the tumor microenvironment, and determining the tumor composition and mutational signatures. This approach covers the precision medicine aspects that have the potential of directing the clinical management of patients with CUP.

In this article, we report a case of CUP diagnosis in a patient with a history of multiple cancer primaries. Despite the histologic similarities with the previously diagnosed urothelial carcinoma, the distinct mutational profile of the metastatic tumor led to a diagnosis of CUP. The lack of response to empirical chemotherapy necessitated further attempts at detailed tumor characterization. Thus, the patient was enrolled in a prospective study to identify the tumor of origin in an attempt to offer a treatment alternative. The BostonGene^©^ Tumor Portrait test was performed on deidentified samples as part of a research collaboration. An integrated tissue of origin WES/RNA-seq analysis ruled out urothelial carcinoma and suggested a lung primary with a high probability. The Tumor Portrait confirmed the high TMB and microenvironment characterized by infiltration by immune cells and a high RNA expression of PD-L1 and PD-1 and a smoking mutational signature. This characterization was needed because even with the identification of the lung primary, empirical treatment could not be offered to this patient with stage IV lung cancer with widespread progressing metastasis and worsening performance status. The patient was treated with ipilimumab + nivolumab because this treatment has been shown to extend survival duration in patients with high TMB, regardless of their PD-L1 status (27). In addition to that, studies have shown that immune infiltration of the tumor is associated with a better response to immune checkpoint inhibitors in general (25). Although it has been reported that RNA-seq PD-L1 and IHC PD-L1 results could be concordant, our case showed a high RNA-seq expression with negative IHC (24). This could be due to unknown technical or biological reasons. However, the high level of expression could be correlated to the response to immune checkpoint inhibitors (28). After three cycles of treatment with ipilimumab + nivolumab, a PET CT scan showed a dramatic radiological response of the primary tumor in the lung and all metastatic tumor lesions in the bone and soft tissue. This report demonstrated that tumor of origin identification using NGS-based tests, like the BostonGene CUP algorithm, could help with improved diagnosis and effective treatment selection. The tumor regression provides evidence that treating CUP patients should go beyond identifying the tumor’s molecular target and origin.

Despite being effective, this method has limitations when managing CUP patients. The availability and quality of the tissue affect the algorithm’s performance and accuracy, similar to other molecular panels. Utilizing FFPE may reduce the DNA and RNA extraction’s quality, which would then make analysis more challenging. Additionally, it may be difficult to biopsy metastatic locations in CUP patients for accurate characterization, as was the case in our patient when the lung lesion could not be biopsied due to extensive emphysema. Despite all the developments, CUP continues to be one of the most challenging cancer types to diagnose and treat. To demonstrate the clinical utility of Tumor Portrait, it must be performed on a large number of CUP patients.

## Data availability statement

The raw data supporting the conclusions of this article is available at (https://www.ncbi.nlm.nih.gov/sra/docs/) via BioProject accession number: PRJNA1063915.

## Ethics statement

The studies involving humans were approved by The Weill Cornell Medicine Institutional Review Board (WCM-IRB). The studies were conducted in accordance with the local legislation and institutional requirements. The participants provided their written informed consent to participate in this study. Written informed consent was obtained from the individual(s) for the publication of any potentially identifiable images or data included in this article.

## Author contributions

MA: Conceptualization, Data curation, Formal analysis, Investigation, Methodology, Writing – original draft, Writing – review & editing. NS: Conceptualization, Data curation, Project administration, Writing – review & editing. MS: Data curation, Funding acquisition, Project administration, Resources, Supervision, Writing – review & editing. JM: Data curation, Investigation, Project administration, Resources, Supervision, Writing – review & editing. ZA: Data curation, Formal analysis, Writing – review & editing. NK: Data curation, Formal analysis, Validation, Writing – review & editing. DK: Data curation, Formal analysis, Writing – review & editing. AN: Data curation, Formal analysis, Writing – review & editing. MK: Data curation, Formal analysis, Writing – review & editing. AT: Data curation, Formal analysis, Writing – review & editing. AM: Data curation, Formal analysis, Writing – review & editing. VF: Data curation, Formal analysis, Writing – review & editing. JD: Data curation, Formal analysis, Writing – review & editing. AB: Data curation, Formal analysis, Writing – review & editing. NA: Data curation, Formal analysis, Project administration, Resources, Supervision, Writing – review & editing. DW: Conceptualization, Investigation, Methodology, Visualization, Writing – review & editing. JE: Formal analysis, Investigation, Validation, Visualization, Writing – review & editing. AS: Data curation, Methodology, Supervision, Validation, Writing – review & editing. OE: Conceptualization, Project administration, Supervision, Writing – review & editing. CS: Conceptualization, Data curation, Formal analysis, Funding acquisition, Investigation, Project administration, Writing – review & editing. DN: Conceptualization, Data curation, Formal analysis, Investigation, Methodology, Project administration, Supervision, Writing – review & editing. JM: Conceptualization, Data curation, Funding acquisition, Investigation, Methodology, Project administration, Resources, Supervision, Validation, Visualization, Writing – review & editing.
